# Acute Renal Failure due to Obstructive Uropathy Secondary to Ureteral Endometriosis

**DOI:** 10.1155/2015/761348

**Published:** 2015-08-09

**Authors:** Jeong In Choi, Jee Geun Yoo, Sa Jin Kim, Hae Nam Lee, Min Jeong Kim

**Affiliations:** Department of Obstetrics and Gynecology, College of Medicine, The Catholic University of Korea, 327 Sosa-ro, Wonmi-gu, Bucheon-si, Gyeonggi-do 420-717, Republic of Korea

## Abstract

Ureteral involvement by endometriosis is a rare and often silent disease but capable of producing significant morbidity and leading to hydronephrosis and to renal failure. Surgery is the treatment of choice to remove endometriotic lesions and relieve ureteral obstruction if the kidney is still functional or a nephrectomy is performed if there is a complete loss of renal function. We report a case of acute renal failure induced ureteral endometriosis managed with laparoscopic unilateral nephrectomy and endometrioma cystectomy. Differential diagnosis is important to confirm diagnosis for patients with ureteral obstruction presenting nonspecific symptoms.

## 1. Introduction

Endometriosis occurs at a high incidence of 15% among reproductive women [1], but it rarely develops into decreased renal function by ureteral endometriosis [2, 3]. Ureteral endometriosis accounts for only a minority of cases (0.1–0.4%) [4], but the incidence is increased to 10–14% in women with rectovaginal endometriotic nodules of more than 3 cm in size [5, 6]. It is usually diagnosed in reproductive women of 30–35 years of age, but it is rare in postmenopausal women [3]. Ureteral endometriosis is usually asymptomatic, but it commonly involves the distal portion of the left ureter in case of extensive endometriosis [1]. Treatment depends on the symptoms of the patient and the degree of renal function maintenance.

We report a case of acute renal failure with hydronephrosis due to obstructive uropathy secondary to ureteral endometriosis, managed with laparoscopic unilateral nephrectomy, endometrioma cystectomy, adhesiolysis, and Double J catheter insertion into the right ureter.

Due to the rare incidence and nonspecific symptoms, ureteral endometriosis should be carefully diagnosed with excluding differential diagnosis and adequately performed prompt treatment.

## 2. Case Presentation

A 45-year-old woman visited our hospital complaining of suddenly developed dysuria, left flank pain, nausea, vomiting, and generalized weakness for one week. She had undergone cesarean section due to fetal breech position at term 10 years ago and the menstrual cycle was regular before hysterectomy. She had been diagnosed with uterine myoma, endometriosis stage IV, and pelvic adhesion and she underwent total abdominal hysterectomy with left salpingectomy due to severe dysmenorrhea 6 years ago.

She was diagnosed with hypertension at a local internal medicine clinic 2 years ago and took medicine (telmisartan; angiotensin II receptor blocker). She had no other underlying history such as diabetes, tuberculosis, and hepatitis.

She had no anemia by initial laboratory tests, and urea nitrogen was 27.0 mg/dL (6.0–20.0), creatinine was 2.57 mg/dL (0.50–1.20), and calculated Fractional Excretion of Sodium (FENa) was 0.47, all of which meant systemic kidney injury. Tumor marker CA 125 was increased to 73.22 U/mL (0–35), and CA19-9 was 35.88 U/mL (0–37).

The 5 cm sized hypoechoic mass was noted in pelvic cavity by two-dimensional transvaginal ultrasound. And renal ultrasound was performed due to complaints of left flank pain, which showed hydronephrosis. As a result, she underwent urologic consultation and pelvic computed tomography (CT) without contrast enhancement.

**Figure 1:**
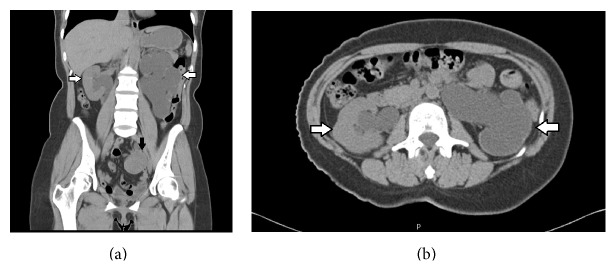
Pelvic CT (computed tomography) showed 4.5 cm sized left adnexal mass (black arrow in (a)) abutting with and obstructing the left distal ureter, resulting in severe left hydroureteronephrosis with complete loss of the left renal parenchyma; it also showed right mild hydroureteronephrosis without definite obstructive lesion (white arrows in (a) and (b)).

**Figure 2:**
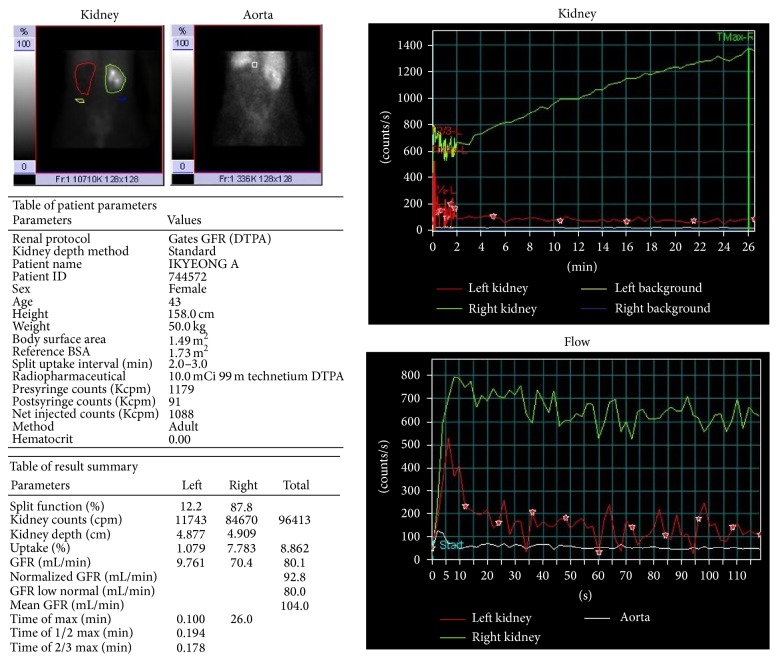
Renal scintigraphy. Complete loss of function of the left kidney.

Pelvic CT without contrast enhancement showed 4.5 cm sized left adnexal mass abutting with and obstructing the left distal ureter (Figure 1(a)), resulting in severe left hydroureteronephrosis with complete loss of the left renal parenchyma. It also showed right mild hydroureteronephrosis without definite obstructive lesion (Figures 1(a) and 1(b)). Renal scintigraphy (Figure 2) showed decreased function of the left kidney of only 12.2% (split function) and confirmed renal failure.

For treatment of acute renal failure, percutaneous nephrotomy of the left kidney was done by radiologic consultation. After one week, urea nitrogen and creatinine improved to 10.9 mg/dL and 1.43 mg/dL, respectively. Since then, pelvic CT with contrast enhancement had been done and confirmed left ovary tumor with obstruction of the left midureter and adherence to the sigmoid colon.

The patient had hydronephrosis with loss of function of the left kidney, left ovary mass suspected ovarian malignancy in pelvic CT, and acute renal failure by blood chemistry; therefore, cooperation was determined by gynecologists and urologists.

In operation, the uterus was not seen due to previous surgery. The right ovary and tube were not identified due to severe bowel adhesion with thick fibrotic tissue, so complete adhesiolysis was impossible. About 5 × 4 cm sized left ovary mass was noted and chocolate colored content was seen. Severe pelvic adhesion was noted and ureterolysis was so difficult; therefore, left nephrectomy was done by retroperitoneal approach and Double J catheter (6 Fr–24 cm) insertion into the right ureter was performed by the urologic team. Complete radical excision was needed but it was limited by severe pelvic adhesion following previous surgery. Instead, we removed all gross lesions and performed maximal adhesiolysis.

The pathologic report confirmed left ovary endometrioma, severe hydroureteronephrosis, and end-stage renal disease of the left kidney.

Postoperative blood chemistry showed that urea nitrogen and creatinine were decreased to 9.3 mg/dL and 1.32 mg/dL, respectively. Pelvic CT showed improvement of previously diagnosed right hydronephrosis, and the patient had no urination difficulty. CA 125 was normalized to 16.12 U/mL and hormonal study for ovarian function showed normal range of follicle stimulating hormone (FSH; 1.66 mIu/mL), luteinizing hormone (LH; 6.56 mIu/mL), and estradiol (E2; 48.56 pg/mL) after 4 months.

She received gonadotropin-releasing hormone (GnRH) agonist treatment for 3 months as a preventive therapy for recurrence of endometriosis. Oral contraceptives were not indicated because she had no uterus. Also she could not receive progesterone therapy because of side effects: edema, epigastric discomfort, and bloating symptoms. Dienogest therapy, emerging treatment of endometriosis recently, was not available at that time in Korea.

Double J catheter of the right ureter was removed 3 months after operation, and blood chemistry performed 12 months later showed maintenance of normal range of urea nitrogen (15.5 mg/dL) and creatinine (1.18 mg/dL).

She had no complications and no evidence of endometriosis recurrence during 3-year follow-up at urology and gynecology outpatient departments and she is free from pain of endometriosis.

## 3. Discussion

Endometriosis is the presence of endometrial tissue outside the uterus; it has been reported to involve the bladder (84%), ureter (10%), kidney (4%), and urethra (2%) [7]. Ureteral endometriosis is often asymmetrical, most frequently involving the distal segment of the left ureter [1], although it can affect both ureters, particularly in patients with extensive pelvic endometriosis [2]. Ureteral endometriosis is usually asymptomatic; however, one-third of the patients have atypical symptoms, such as dysmenorrhea, dyspareunia, pelvic pain, infertility, urinary frequency, recurrent urinary tract infection, and back pain [2]. Rarely, hematuria, hypertension, and acute renal failure [2, 8] are also observed.

Urogenital systemic involvement by endometriosis is not an independent disease; it is usually accompanied by pelvic endometriosis, and our patient also had a history of previous surgery with severe pelvic endometriosis.

Ureteral involvement by endometriosis can cause ureteral obstruction and lead to hydroureteronephrosis, and it can develop loss of renal function [2]. Most of the patients show hydroureter and hydronephrosis; 33% of them have pyelonephritis, and 30% have decreased renal function when confirming diagnosis [2]. Donnez et al. reported the risk of loss of renal function with 11.5% of ureteral endometriosis patients [5]. Up to 47% of the patients will require nephrectomy at the time of diagnosis for nonfunctioning kidney or ureteral endometrial lesion mimicking transitional cell carcinoma [9].

Since there are no particular urologic symptoms, it is difficult to find ureteral endometriosis by preoperative evaluations or radiologic studies. Our patient had nonspecific symptoms such as dysuria, left flank pain, nausea, vomiting, and generalized weakness and confirmed hydroureteronephrosis by the radiologic study. After laboratory test, she was diagnosed with acute renal failure and operation was determined due to suspected ovarian tumor and nonfunctioning left kidney.

Various methods of investigation such as intravenous pyelography (IVP), retrograde pyelography (RGP), ultrasound, kidney scintigraphy, and magnetic resonance imaging (MRI) have been proposed in cases of clinical suspicion of ureteral endometriosis [1]. Ultrasound is a good screening test for pelvic endometriosis [10], but it cannot be found in case of endometriosis in ureteral parenchyma without dilatation of ureter [1]. The best diagnostic test is IVP, which can be confirmed by a filling defect of contrast within the lumen of the ureter [1]. IVP is necessary in patients with rectovaginal endometriotic nodules of more than 3 cm in size, if they have no typical symptoms of hydroureteronephrosis. When hydroureteronephrosis is confirmed by IVP, renal scintigraphy should be performed to test the renal function [11]. MRI is a sensitive, specific [2], and effective diagnostic tool for predicting operative findings; but it is a very expensive test to perform on every patient. Final diagnosis should be confirmed by pathologic examination.

The management of ureteral endometriosis depends on the extent of the disease and the degree of renal function compromise due to ureteral involvement, as well as the extent of pelvic and urologic disease and the severity of pain [1]. The treatment options for ureteral endometriosis include hormonal therapy, surgical therapy, or a combination of both. Conservative procedures such as ureteral stenting, associated with medical treatment, usually lead to favorable outcomes [12]. Surgery is the treatment of choice with advanced disease. Proposed surgical interventions for relief of obstructive uropathy caused by endometrial tissue include ureterolysis, distal ureterectomy, and ureteral reimplantation or interposition of an ileal segment between the ureter and bladder [13]. End-to-end anastomosis can be performed after ureterectomy or incision of distal ureter [11]. Complete loss of renal function is an indication of nephrectomy, as a nonfunctioning kidney associated hydronephrosis can lead to vascular hypertension, recurrent pyelonephritis, or kidney stones [11]. Nephroureterectomy is a successful treatment alternative in cases of refractory pain in patients with ureteral endometriosis.

It is difficult to diagnose and treat ureteral endometriosis; treatment should be started after multidisciplinary approach and consultations, and gross lesions should be removed during operation. Furthermore, the degree of renal function maintenance should be closely checked for treatment policy determination.

Oral contraceptives were not indicated because she had no uterus. Also she could not receive progesterone therapy because of side effects: edema, epigastric discomfort, and bloating symptoms. Dienogest therapy, emerging treatment of endometriosis recently, was not available at that time in Korea.

We report a case of acute renal failure with hydronephrosis due to obstructive uropathy secondary to ureteral endometriosis, managed with laparoscopic unilateral nephrectomy, endometrioma cystectomy, adhesiolysis, and Double J catheter insertion into the right ureter. The patient received GnRH agonist treatment for 3 months as a preventive therapy for recurrence of endometriosis, and her right renal function is well-maintained.

Differential diagnosis is important to confirm diagnosis for patients with ureteral obstruction presenting nonspecific symptoms. Although the occurrence of acute renal failure induced by endometriosis is very rare, endometriosis should be considered as a differential diagnosis for these patients. Complete examination including laboratory tests and radiologic examinations should be performed to differentiate from other diagnoses and treat patients adequately.
